# Supplementary figures and images for: Predictive representations can link model-based reinforcement learning to model-free mechanisms
Source: PLoS Comput Biol. 2017 Sep 25;13(9):e1005768. doi: 10.1371/journal.pcbi.1005768 (PMC5628940; doi:10.1371/journal.pcbi.1005768)

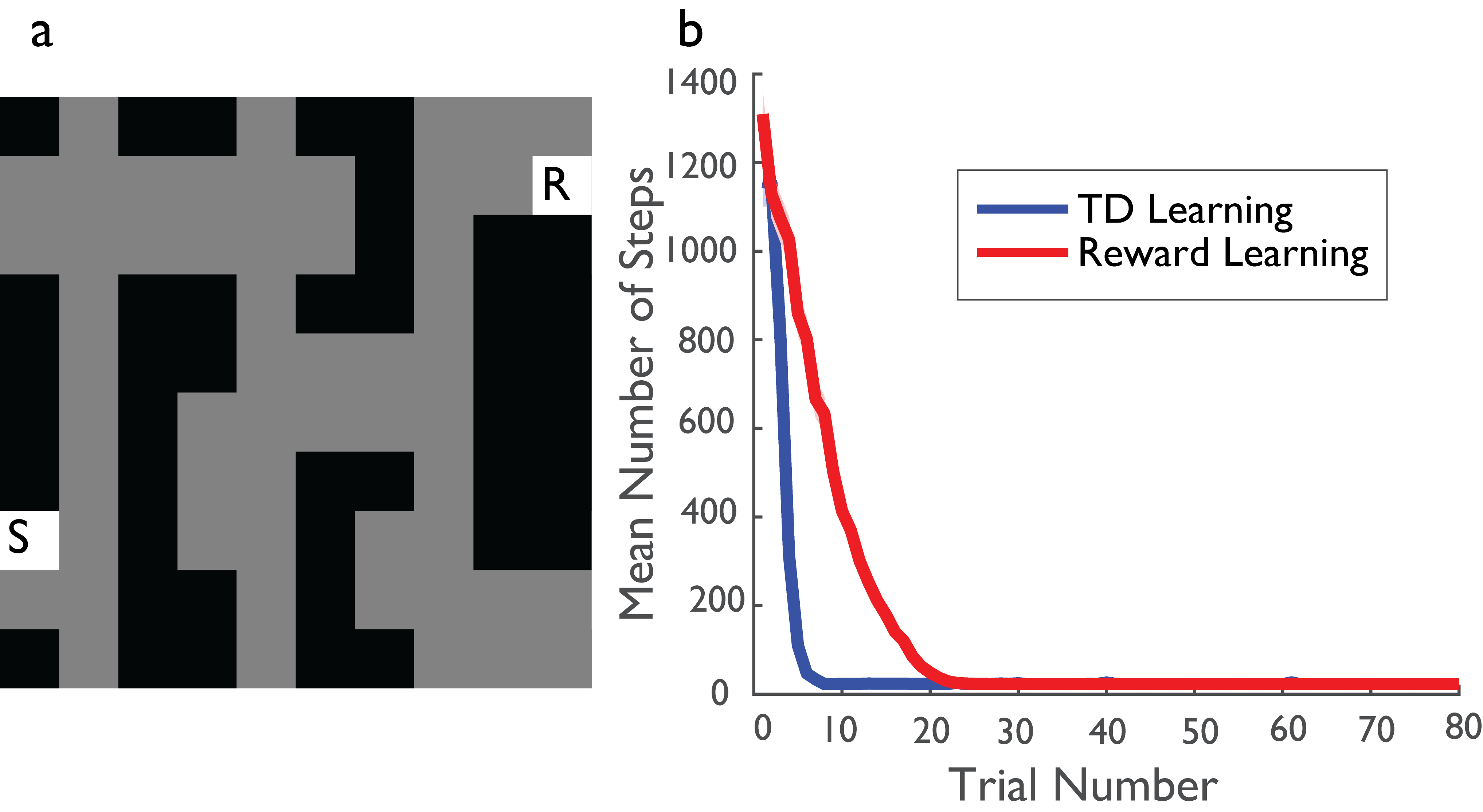

Supplement: S1 Fig — a) Task environment. On each trial, the agent was placed in state S. Trials ended when the agent reached state R, which contained a reward value of 10. Unlike the latent learning task in the main text, this task did not contain an exploratory period enabling the agent to learn the successor matrix prior to the introduction of reward. b) Number of steps on each trial for an agent learning weights using TD learning and an agent learning weights applying delta rule to the reward function. Plotted lines show average over 500 runs. 95% confidence intervals are contained within line thickness. Parameters for each of the two algorithms were set to those that minimized the average number of total steps over 80 trials. Such parameters were found by grid search over αsr ∈ [.1,.3,.5,.7,.9], ϵ ∈ [0.1,0.3,0.5] and αw ∈ [.1,.3,.5,.7,.9]. Both algorithms learned the SR using the SR-TD update. The “TD Learning” algorithm updated weights using TD learning. The “Reward Learning” algorithm updated weights by delta-rule learning on the immediate reward function. Specifically, after performing action a in state s and receiving reward r, the following update was performed: w(s) ← w(s) + αw(r − w(s)).” (TIF) [file pcbi.1005768.s001.tif]

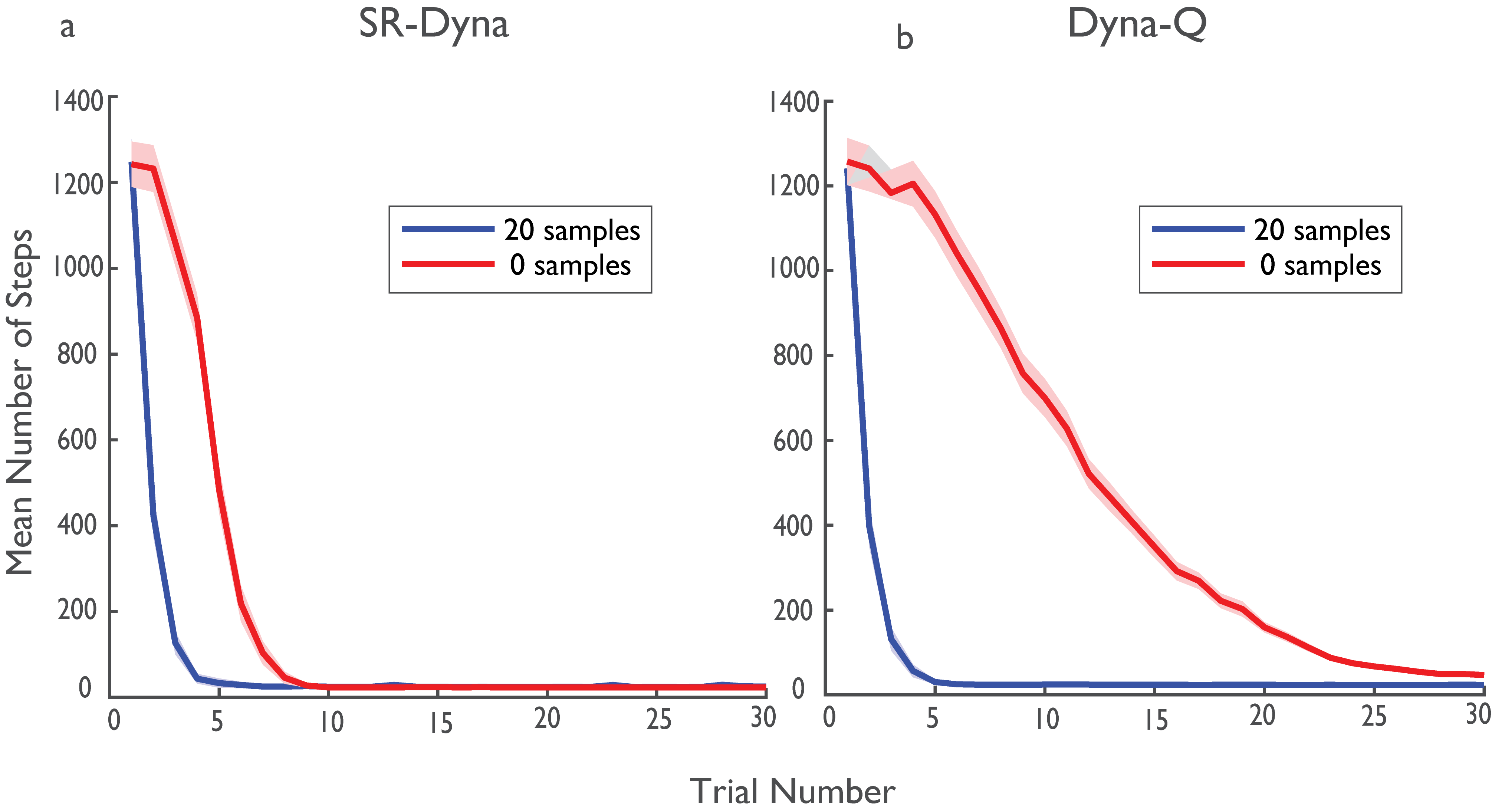

Supplement: S2 Fig — Both algorithms under the two sampling settings were simulated on the task displayed in S1 Fig. a) Results of simulations with SR-Dyna. b) Results of simulations with Dyna-Q. Both a) and b) show number of steps on each trial for agent permitted to replay 20 samples between each decision and an agent not permitted to replay any samples. Plotted lines show average over 500 runs. 95% confidence intervals are contained within shaded region around lines. For each algorithm and sample setting, we chose parameters that minimized average total number of steps over 80 trials by a grid search in the following range: αsr ∈ [.1,.3,.5,.7,.9], ϵ ∈ [0.1,0.3,0.5], αw ∈ [.1,.3,.5,.7,.9], and αQ ∈ [.1,.3,.5,.7,.9]. (TIF) [file pcbi.1005768.s002.tif]
