# Supplementary material for: Predictive representations can link model-based reinforcement learning to model-free mechanisms
Source: PLoS Comput Biol. 2017 Sep 25;13(9):e1005768. doi: 10.1371/journal.pcbi.1005768 (PMC5628940; doi:10.1371/journal.pcbi.1005768)
Supplement: S1 Table — Here, we display the results of simulating each task, using each algorithm under a wide variety of parameter settings. Each table below corresponds to a particular algorithm simulating a particular task. For a given parameter setting, the algorithm was simulated 500 times. A check indicates that the 500 run median value function produced by that parameter setting results in the optimal policy for the task. A cross indicates that it does not result in the optimal policy. (DOCX) [file pcbi.1005768.s003.docx]

**Latent learning task**

*SR-TD*

|  | $\epsilon=0.1$ | | | | | | $\epsilon=0.3$ | | | | | $\epsilon=0.5$ | | | | |
| --- | --- | --- | --- | --- | --- | --- | --- | --- | --- | --- | --- | --- | --- | --- | --- | --- |
|  | $\alpha_{w}$ | | | | | | $\alpha_{w}$ | | | | | $\alpha_{w}$ | | | | |
| $\alpha_{sr}$ |  | 0.1 | 0.3 | 0.5 | 0.7 | 0.9 | 0.1 | 0.3 | 0.5 | 0.7 | 0.9 | 0.1 | 0.3 | 0.5 | 0.7 | 0.9 |
|  | 0.1 | ✔ | ✔ | ✔ | ✔ | ✔ | ✔ | ✔ | ✔ | ✔ | ✔ | ✔ | ✔ | ✔ | ✔ | ✔ |
|  | 0.3 | ✔ | ✔ | ✔ | ✔ | ✔ | ✔ | ✔ | ✔ | ✔ | ✔ | ✔ | ✔ | ✔ | ✔ | ✔ |
|  | 0.5 | ✔ | ✔ | ✔ | ✔ | ✔ | ✔ | ✔ | ✔ | ✔ | ✔ | ✔ | ✔ | ✔ | ✔ | ✔ |
|  | 0.7 | ✔ | ✔ | ✔ | ✔ | ✔ | ✔ | ✔ | ✔ | ✔ | ✔ | ✔ | ✔ | ✔ | ✔ | ✔ |
|  | 0.9 | ✔ | ✔ | ✔ | ✔ | ✔ | ✔ | ✔ | ✔ | ✔ | ✔ | ✔ | ✔ | ✔ | ✔ | ✔ |

*SR-MB*

|  | $\epsilon=0.1$ | | | | | | $\epsilon=0.3$ | | | | | $\epsilon=0.5$ | | | | |
| --- | --- | --- | --- | --- | --- | --- | --- | --- | --- | --- | --- | --- | --- | --- | --- | --- |
|  | $\alpha_{w}$ | | | | | | $\alpha_{w}$ | | | | | $\alpha_{w}$ | | | | |
| $\alpha_{\pi}$ |  | 0.1 | 0.3 | 0.5 | 0.7 | 0.9 | 0.1 | 0.3 | 0.5 | 0.7 | 0.9 | 0.1 | 0.3 | 0.5 | 0.7 | 0.9 |
|  | 0.1 | ✔ | ✔ | ✔ | ✔ | ✔ | ✔ | ✔ | ✔ | ✔ | ✔ | ✔ | ✔ | ✔ | ✔ | ✔ |
|  | 0.3 | ✔ | ✔ | ✔ | ✔ | ✔ | ✔ | ✔ | ✔ | ✔ | ✔ | ✔ | ✔ | ✔ | ✔ | ✔ |
|  | 0.5 | ✔ | ✔ | ✔ | ✔ | ✔ | ✔ | ✔ | ✔ | ✔ | ✔ | ✔ | ✔ | ✔ | ✔ | ✔ |
|  | 0.7 | ✔ | ✔ | ✔ | ✔ | ✔ | ✔ | ✔ | ✔ | ✔ | ✔ | ✔ | ✔ | ✔ | ✔ | ✔ |
|  | 0.9 | ✖ | ✔ | ✔ | ✖ | ✖ | ✔ | ✖ | ✖ | ✖ | ✔ | ✔ | ✖ | ✔ | ✖ | ✔ |

*SR-dyna, k = 10000*

|  | $\epsilon=0.1$ | | | | | | $\epsilon=0.3$ | | | | | $\epsilon=0.5$ | | | | |
| --- | --- | --- | --- | --- | --- | --- | --- | --- | --- | --- | --- | --- | --- | --- | --- | --- |
|  | $\alpha_{w}$ | | | | | | $\alpha_{w}$ | | | | | $\alpha_{w}$ | | | | |
| $\alpha_{sr}$ |  | 0.1 | 0.3 | 0.5 | 0.7 | 0.9 | 0.1 | 0.3 | 0.5 | 0.7 | 0.9 | 0.1 | 0.3 | 0.5 | 0.7 | 0.9 |
|  | 0.1 | ✔ | ✔ | ✔ | ✔ | ✔ | ✔ | ✔ | ✔ | ✔ | ✔ | ✔ | ✔ | ✔ | ✔ | ✔ |
|  | 0.3 | ✔ | ✔ | ✔ | ✔ | ✔ | ✔ | ✔ | ✔ | ✔ | ✔ | ✔ | ✔ | ✔ | ✔ | ✔ |
|  | 0.5 | ✔ | ✔ | ✔ | ✔ | ✔ | ✔ | ✔ | ✔ | ✔ | ✔ | ✔ | ✔ | ✔ | ✔ | ✔ |
|  | 0.7 | ✔ | ✔ | ✔ | ✔ | ✔ | ✔ | ✔ | ✔ | ✔ | ✔ | ✔ | ✔ | ✔ | ✔ | ✔ |
|  | 0.9 | ✔ | ✔ | ✔ | ✔ | ✔ | ✔ | ✔ | ✔ | ✔ | ✔ | ✔ | ✔ | ✔ | ✔ | ✔ |

*SR-dyna, k = 10*

|  | $\epsilon=0.1$ | | | | | | $\epsilon=0.3$ | | | | | $\epsilon=0.5$ | | | | |
| --- | --- | --- | --- | --- | --- | --- | --- | --- | --- | --- | --- | --- | --- | --- | --- | --- |
|  | $\alpha_{w}$ | | | | | | $\alpha_{w}$ | | | | | $\alpha_{w}$ | | | | |
| $\alpha_{sr}$ |  | 0.1 | 0.3 | 0.5 | 0.7 | 0.9 | 0.1 | 0.3 | 0.5 | 0.7 | 0.9 | 0.1 | 0.3 | 0.5 | 0.7 | 0.9 |
|  | 0.1 | ✔ | ✔ | ✔ | ✔ | ✔ | ✔ | ✔ | ✔ | ✔ | ✔ | ✔ | ✔ | ✔ | ✔ | ✔ |
|  | 0.3 | ✔ | ✔ | ✔ | ✔ | ✔ | ✔ | ✔ | ✔ | ✔ | ✔ | ✔ | ✔ | ✔ | ✔ | ✔ |
|  | 0.5 | ✔ | ✔ | ✔ | ✔ | ✔ | ✔ | ✔ | ✔ | ✔ | ✔ | ✔ | ✔ | ✔ | ✔ | ✔ |
|  | 0.7 | ✔ | ✔ | ✔ | ✔ | ✔ | ✔ | ✔ | ✔ | ✔ | ✔ | ✔ | ✔ | ✔ | ✔ | ✔ |
|  | 0.9 | ✔ | ✔ | ✔ | ✔ | ✔ | ✔ | ✔ | ✔ | ✔ | ✔ | ✔ | ✔ | ✔ | ✔ | ✔ |

*Dyna-Q, k = 10000*

|  |  | $\epsilon=0.1$ | $\epsilon=0.3$ | $\epsilon=0.5$ |
| --- | --- | --- | --- | --- |
| $\alpha_{Q}$ | 0.1 | ✔ | ✔ | ✔ |
|  | 0.3 | ✔ | ✔ | ✔ |
|  | 0.5 | ✔ | ✔ | ✔ |
|  | 0.7 | ✔ | ✔ | ✔ |
|  | 0.9 | ✔ | ✔ | ✔ |

*Dyna-Q, k = 10*

|  |  | $\epsilon=0.1$ | $\epsilon=0.3$ | $\epsilon=0.5$ |
| --- | --- | --- | --- | --- |
| $\alpha_{Q}$ | 0.1 | ✖ | ✖ | ✖ |
|  | 0.3 | ✖ | ✖ | ✖ |
|  | 0.5 | ✖ | ✖ | ✖ |
|  | 0.7 | ✖ | ✖ | ✖ |
|  | 0.9 | ✖ | ✖ | ✖ |

*1-step lookahead*

|  |  | $\epsilon=0.1$ | $\epsilon=0.3$ | $\epsilon=0.5$ |
| --- | --- | --- | --- | --- |
| $\alpha_{V}$ | 0.1 | ✖ | ✖ | ✖ |
|  | 0.3 | ✖ | ✖ | ✖ |
|  | 0.5 | ✖ | ✖ | ✖ |
|  | 0.7 | ✖ | ✖ | ✖ |
|  | 0.9 | ✖ | ✖ | ✖ |

**Detour Task**

SR-TD

|  | $\epsilon=0.1$ | | | | | | $\epsilon=0.3$ | | | | | $\epsilon=0.5$ | | | | |
| --- | --- | --- | --- | --- | --- | --- | --- | --- | --- | --- | --- | --- | --- | --- | --- | --- |
|  | $\alpha_{w}$ | | | | | | $\alpha_{w}$ | | | | | $\alpha_{w}$ | | | | |
| $\alpha_{sr}$ |  | 0.1 | 0.3 | 0.5 | 0.7 | 0.9 | 0.1 | 0.3 | 0.5 | 0.7 | 0.9 | 0.1 | 0.3 | 0.5 | 0.7 | 0.9 |
|  | 0.1 | ✖ | ✖ | ✖ | ✖ | ✖ | ✖ | ✖ | ✖ | ✖ | ✖ | ✖ | ✖ | ✖ | ✖ | ✖ |
|  | 0.3 | ✖ | ✖ | ✖ | ✖ | ✖ | ✖ | ✖ | ✖ | ✖ | ✖ | ✖ | ✖ | ✖ | ✖ | ✖ |
|  | 0.5 | ✖ | ✖ | ✖ | ✖ | ✖ | ✖ | ✖ | ✖ | ✖ | ✖ | ✖ | ✖ | ✖ | ✖ | ✖ |
|  | 0.7 | ✖ | ✖ | ✖ | ✖ | ✖ | ✖ | ✖ | ✖ | ✖ | ✖ | ✖ | ✖ | ✖ | ✖ | ✖ |
|  | 0.9 | ✖ | ✖ | ✖ | ✖ | ✖ | ✖ | ✖ | ✖ | ✖ | ✖ | ✖ | ✖ | ✖ | ✖ | ✖ |

SR-MB

|  | $\epsilon=0.1$ | | | | | | $\epsilon=0.3$ | | | | | $\epsilon=0.5$ | | | | |
| --- | --- | --- | --- | --- | --- | --- | --- | --- | --- | --- | --- | --- | --- | --- | --- | --- |
|  | $\alpha_{w}$ | | | | | | $\alpha_{w}$ | | | | | $\alpha_{w}$ | | | | |
| $\alpha_{\pi}$ |  | 0.1 | 0.3 | 0.5 | 0.7 | 0.9 | 0.1 | 0.3 | 0.5 | 0.7 | 0.9 | 0.1 | 0.3 | 0.5 | 0.7 | 0.9 |
|  | 0.1 | ✔ | ✔ | ✔ | ✔ | ✔ | ✔ | ✔ | ✔ | ✔ | ✔ | ✔ | ✔ | ✔ | ✔ | ✔ |
|  | 0.3 | ✔ | ✔ | ✔ | ✔ | ✔ | ✔ | ✔ | ✔ | ✔ | ✔ | ✔ | ✔ | ✔ | ✔ | ✔ |
|  | 0.5 | ✔ | ✔ | ✔ | ✔ | ✔ | ✔ | ✔ | ✔ | ✔ | ✔ | ✔ | ✔ | ✔ | ✔ | ✔ |
|  | 0.7 | ✔ | ✔ | ✔ | ✔ | ✖ | ✔ | ✔ | ✔ | ✔ | ✔ | ✔ | ✔ | ✔ | ✔ | ✔ |
|  | 0.9 | ✔ | ✔ | ✔ | ✖ | ✖ | ✔ | ✔ | ✔ | ✖ | ✔ | ✔ | ✔ | ✔ | ✖ | ✖ |

*SR-dyna high*

|  | $\epsilon=0.1$ | | | | | | $\epsilon=0.3$ | | | | | $\epsilon=0.5$ | | | | |
| --- | --- | --- | --- | --- | --- | --- | --- | --- | --- | --- | --- | --- | --- | --- | --- | --- |
|  | $\alpha_{w}$ | | | | | | $\alpha_{w}$ | | | | | $\alpha_{w}$ | | | | |
| $\alpha_{sr}$ |  | 0.1 | 0.3 | 0.5 | 0.7 | 0.9 | 0.1 | 0.3 | 0.5 | 0.7 | 0.9 | 0.1 | 0.3 | 0.5 | 0.7 | 0.9 |
|  | 0.1 | ✔ | ✖ | ✖ | ✖ | ✖ | ✔ | ✔ | ✔ | ✔ | ✔ | ✔ | ✔ | ✔ | ✔ | ✖ |
|  | 0.3 | ✔ | ✔ | ✖ | ✖ | ✖ | ✔ | ✔ | ✔ | ✖ | ✔ | ✔ | ✔ | ✔ | ✔ | ✔ |
|  | 0.5 | ✔ | ✔ | ✔ | ✔ | ✔ | ✔ | ✔ | ✔ | ✖ | ✔ | ✔ | ✔ | ✔ | ✔ | ✔ |
|  | 0.7 | ✔ | ✔ | ✔ | ✔ | ✔ | ✔ | ✔ | ✔ | ✖ | ✖ | ✔ | ✔ | ✖ | ✖ | ✖ |
|  | 0.9 | ✔ | ✔ | ✔ | ✔ | ✖ | ✔ | ✔ | ✔ | ✖ | ✖ | ✔ | ✔ | ✔ | ✖ | ✖ |

*SR-dyna-low*

|  | $\epsilon=0.1$ | | | | | | $\epsilon=0.3$ | | | | | $\epsilon=0.5$ | | | | |
| --- | --- | --- | --- | --- | --- | --- | --- | --- | --- | --- | --- | --- | --- | --- | --- | --- |
|  | $\alpha_{w}$ | | | | | | $\alpha_{w}$ | | | | | $\alpha_{w}$ | | | | |
| $\alpha_{sr}$ |  | 0.1 | 0.3 | 0.5 | 0.7 | 0.9 | 0.1 | 0.3 | 0.5 | 0.7 | 0.9 | 0.1 | 0.3 | 0.5 | 0.7 | 0.9 |
|  | 0.1 | ✖ | ✖ | ✖ | ✖ | ✖ | ✖ | ✖ | ✖ | ✖ | ✖ | ✖ | ✖ | ✖ | ✖ | ✖ |
|  | 0.3 | ✖ | ✖ | ✖ | ✖ | ✖ | ✖ | ✖ | ✖ | ✖ | ✖ | ✖ | ✖ | ✖ | ✖ | ✖ |
|  | 0.5 | ✖ | ✖ | ✖ | ✖ | ✖ | ✖ | ✖ | ✖ | ✖ | ✖ | ✖ | ✖ | ✖ | ✖ | ✖ |
|  | 0.7 | ✖ | ✖ | ✖ | ✖ | ✖ | ✖ | ✖ | ✖ | ✖ | ✖ | ✖ | ✖ | ✖ | ✖ | ✖ |
|  | 0.9 | ✖ | ✖ | ✖ | ✖ | ✖ | ✖ | ✖ | ✖ | ✖ | ✖ | ✖ | ✖ | ✖ | ✖ | ✖ |

*Dyna-Q high*

|  |  | $\epsilon=0.1$ | $\epsilon=0.3$ | $\epsilon=0.5$ |
| --- | --- | --- | --- | --- |
| $\alpha_{Q}$ | 0.1 | ✔ | ✔ | ✔ |
|  | 0.3 | ✔ | ✔ | ✔ |
|  | 0.5 | ✔ | ✔ | ✔ |
|  | 0.7 | ✔ | ✔ | ✔ |
|  | 0.9 | ✔ | ✔ | ✔ |

*Dyna-Q low*

|  |  | $\epsilon=0.1$ | $\epsilon=0.3$ | $\epsilon=0.5$ |
| --- | --- | --- | --- | --- |
| $\alpha_{Q}$ | 0.1 | ✖ | ✖ | ✖ |
|  | 0.3 | ✖ | ✖ | ✖ |
|  | 0.5 | ✖ | ✖ | ✖ |
|  | 0.7 | ✖ | ✖ | ✖ |
|  | 0.9 | ✖ | ✖ | ✖ |

*1-step lookahead*

|  |  | $\epsilon=0.1$ | $\epsilon=0.3$ | $\epsilon=0.5$ |
| --- | --- | --- | --- | --- |
| $\alpha_{V}$ | 0.1 | ✖ | ✖ | ✖ |
|  | 0.3 | ✖ | ✖ | ✖ |
|  | 0.5 | ✖ | ✖ | ✖ |
|  | 0.7 | ✖ | ✖ | ✖ |
|  | 0.9 | ✖ | ✖ | ✖ |

**Policy revaluation task**

SR-TD

|  | $\epsilon=0.1$ | | | | | | $\epsilon=0.3$ | | | | | $\epsilon=0.5$ | | | | |
| --- | --- | --- | --- | --- | --- | --- | --- | --- | --- | --- | --- | --- | --- | --- | --- | --- |
|  | $\alpha_{w}$ | | | | | | $\alpha_{w}$ | | | | | $\alpha_{w}$ | | | | |
| $\alpha_{sr}$ |  | 0.1 | 0.3 | 0.5 | 0.7 | 0.9 | 0.1 | 0.3 | 0.5 | 0.7 | 0.9 | 0.1 | 0.3 | 0.5 | 0.7 | 0.9 |
|  | 0.1 | ✖ | ✖ | ✖ | ✖ | ✖ | ✖ | ✖ | ✖ | ✖ | ✖ | ✖ | ✖ | ✖ | ✖ | ✖ |
|  | 0.3 | ✖ | ✖ | ✖ | ✖ | ✖ | ✖ | ✖ | ✖ | ✖ | ✖ | ✖ | ✖ | ✖ | ✖ | ✖ |
|  | 0.5 | ✖ | ✖ | ✖ | ✖ | ✖ | ✖ | ✖ | ✖ | ✖ | ✖ | ✖ | ✖ | ✖ | ✖ | ✖ |
|  | 0.7 | ✖ | ✖ | ✖ | ✖ | ✖ | ✖ | ✖ | ✖ | ✖ | ✖ | ✖ | ✖ | ✖ | ✖ | ✖ |
|  | 0.9 | ✖ | ✖ | ✖ | ✖ | ✖ | ✖ | ✖ | ✖ | ✖ | ✖ | ✖ | ✖ | ✖ | ✖ | ✖ |

SR-MB

|  | $\epsilon=0.1$ | | | | | | $\epsilon=0.3$ | | | | | $\epsilon=0.5$ | | | | |
| --- | --- | --- | --- | --- | --- | --- | --- | --- | --- | --- | --- | --- | --- | --- | --- | --- |
|  | $\alpha_{w}$ | | | | | | $\alpha_{w}$ | | | | | $\alpha_{w}$ | | | | |
| $\alpha_{\pi}$ |  | 0.1 | 0.3 | 0.5 | 0.7 | 0.9 | 0.1 | 0.3 | 0.5 | 0.7 | 0.9 | 0.1 | 0.3 | 0.5 | 0.7 | 0.9 |
|  | 0.1 | ✖ | ✖ | ✖ | ✖ | ✖ | ✖ | ✖ | ✖ | ✖ | ✖ | ✖ | ✖ | ✖ | ✖ | ✖ |
|  | 0.3 | ✖ | ✖ | ✖ | ✖ | ✖ | ✖ | ✖ | ✖ | ✖ | ✖ | ✖ | ✖ | ✖ | ✖ | ✖ |
|  | 0.5 | ✖ | ✖ | ✖ | ✖ | ✖ | ✖ | ✖ | ✖ | ✖ | ✖ | ✖ | ✖ | ✖ | ✖ | ✖ |
|  | 0.7 | ✖ | ✖ | ✖ | ✖ | ✖ | ✖ | ✖ | ✖ | ✖ | ✖ | ✖ | ✖ | ✖ | ✖ | ✖ |
|  | 0.9 | ✖ | ✖ | ✖ | ✖ | ✖ | ✖ | ✖ | ✖ | ✖ | ✖ | ✖ | ✖ | ✖ | ✖ | ✖ |

*SR-dyna high*

|  | $\epsilon=0.1$ | | | | | | $\epsilon=0.3$ | | | | | $\epsilon=0.5$ | | | | |
| --- | --- | --- | --- | --- | --- | --- | --- | --- | --- | --- | --- | --- | --- | --- | --- | --- |
|  | $\alpha_{w}$ | | | | | | $\alpha_{w}$ | | | | | $\alpha_{w}$ | | | | |
| $\alpha_{sr}$ |  | 0.1 | 0.3 | 0.5 | 0.7 | 0.9 | 0.1 | 0.3 | 0.5 | 0.7 | 0.9 | 0.1 | 0.3 | 0.5 | 0.7 | 0.9 |
|  | 0.1 | ✔ | ✔ | ✔ | ✔ | ✔ | ✔ | ✔ | ✔ | ✔ | ✔ | ✔ | ✔ | ✔ | ✔ | ✔ |
|  | 0.3 | ✔ | ✔ | ✔ | ✔ | ✔ | ✔ | ✔ | ✔ | ✔ | ✔ | ✔ | ✔ | ✔ | ✔ | ✔ |
|  | 0.5 | ✔ | ✔ | ✔ | ✔ | ✔ | ✔ | ✔ | ✔ | ✔ | ✔ | ✔ | ✔ | ✔ | ✔ | ✔ |
|  | 0.7 | ✔ | ✔ | ✔ | ✔ | ✔ | ✔ | ✔ | ✔ | ✔ | ✔ | ✔ | ✔ | ✔ | ✔ | ✔ |
|  | 0.9 | ✔ | ✔ | ✔ | ✔ | ✔ | ✔ | ✔ | ✔ | ✔ | ✔ | ✔ | ✔ | ✔ | ✔ | ✔ |

*SR-dyna low*

|  | $\epsilon=0.1$ | | | | | | $\epsilon=0.3$ | | | | | $\epsilon=0.5$ | | | | |
| --- | --- | --- | --- | --- | --- | --- | --- | --- | --- | --- | --- | --- | --- | --- | --- | --- |
|  | $\alpha_{w}$ | | | | | | $\alpha_{w}$ | | | | | $\alpha_{w}$ | | | | |
| $\alpha_{sr}$ |  | 0.1 | 0.3 | 0.5 | 0.7 | 0.9 | 0.1 | 0.3 | 0.5 | 0.7 | 0.9 | 0.1 | 0.3 | 0.5 | 0.7 | 0.9 |
|  | 0.1 | ✖ | ✖ | ✖ | ✖ | ✖ | ✖ | ✖ | ✖ | ✖ | ✖ | ✖ | ✖ | ✖ | ✖ | ✖ |
|  | 0.3 | ✖ | ✖ | ✖ | ✖ | ✖ | ✖ | ✖ | ✖ | ✖ | ✖ | ✖ | ✖ | ✖ | ✖ | ✖ |
|  | 0.5 | ✖ | ✖ | ✖ | ✖ | ✖ | ✖ | ✖ | ✖ | ✖ | ✖ | ✖ | ✖ | ✖ | ✖ | ✖ |
|  | 0.7 | ✖ | ✖ | ✖ | ✖ | ✖ | ✖ | ✖ | ✖ | ✖ | ✖ | ✖ | ✖ | ✖ | ✖ | ✖ |
|  | 0.9 | ✖ | ✖ | ✖ | ✖ | ✖ | ✖ | ✖ | ✖ | ✖ | ✖ | ✖ | ✖ | ✖ | ✖ | ✖ |

*Dyna-Q high*

|  |  | $\epsilon=0.1$ | $\epsilon=0.3$ | $\epsilon=0.5$ |
| --- | --- | --- | --- | --- |
| $\alpha_{Q}$ | 0.1 | ✔ | ✔ | ✔ |
|  | 0.3 | ✔ | ✔ | ✔ |
|  | 0.5 | ✔ | ✔ | ✔ |
|  | 0.7 | ✔ | ✔ | ✔ |
|  | 0.9 | ✔ | ✔ | ✔ |

*Dyna-Q low*

|  |  | $\epsilon=0.1$ | $\epsilon=0.3$ | $\epsilon=0.5$ |
| --- | --- | --- | --- | --- |
| $\alpha_{Q}$ | 0.1 | ✖ | ✖ | ✖ |
|  | 0.3 | ✖ | ✖ | ✖ |
|  | 0.5 | ✖ | ✖ | ✖ |
|  | 0.7 | ✖ | ✖ | ✖ |
|  | 0.9 | ✖ | ✖ | ✖ |

*1-step lookahead*

|  |  | $\epsilon=0.1$ | $\epsilon=0.3$ | $\epsilon=0.5$ |
| --- | --- | --- | --- | --- |
| $\alpha_{V}$ | 0.1 | ✖ | ✖ | ✖ |
|  | 0.3 | ✖ | ✖ | ✖ |
|  | 0.5 | ✖ | ✖ | ✖ |
|  | 0.7 | ✖ | ✖ | ✖ |
|  | 0.9 | ✖ | ✖ | ✖ |
